# H9N2 avian influenza infection altered expression pattern of Sphiogosine-1-phosphate Receptor 1 in BALB/c mice

**DOI:** 10.1186/1743-422X-10-296

**Published:** 2013-09-30

**Authors:** Shuang Tong, Jin Tian, Heng Wang, Zhiqiang Huang, Meng Yu, Lingshuang Sun, Rongchang Liu, Ming Liao, Zhangyong Ning

**Affiliations:** 1 College of Veterinary Medicine, South China Agricultural University, Guangzhou 510642, People’s Republic of China

**Keywords:** H9N2, BALB/c mice, S1PR1, Expression pattern

## Abstract

**Background:**

The pathological damage inflicted by virulent AIV strains is often caused by inducing a positive feedback loop of cytokines in immune cells that cause excessive inflammation. Previous research has shown that a G protein-coupled receptor, sphingosine-1-phosphate receptor 1 (S1PR1), plays a crucial role in the development of excessive inflammation in influenza virus infection (Cell 146:861–862, 2011; Cell 146:980–991, 2011). BALB/c mice are common laboratory animals used in research of influenza virus; however the effects of influenza infections on expression patterns of S1PR1 in mice are unknown.

**Methods:**

We investigated the expression patterns of S1PR1 in normal BALB/c mice and those infected by two distinct H9N2 AIV strains, one (A/chicken/Guangdong/V/2008,V) highly pathogenic, and the other (A/chicken/Guangdong/Ts/2004,Ts), non-pathogenic in mice, using quantitative PCR and immunohistochemistry (IHC) to detect S1PR1 mRNA and protein, respectively.

**Results:**

S1PR1 mRNA was ubiquitously expressed in all the tissues examined, and significant differences were seen in mRNA expression between infected Ts, V and control mice in detected tissues, heart, liver, spleen, kidney and brain. S1PR1 protein was expressed in the cytoplasm and also demonstrated quantitative changes in expression in the various tissues between mice infected with the two strains of AIV.

**Conclusions:**

Our results provided the first look at differences in S1PR1 expression patterns in BALB/c mice infected by non-pathogenic and highly pathogenic H9N2 influenza viruses. This information will not only be helpful in designing experiments to better understand the role of S1PR1 in virus-host interactions but also in developing novel anti-influenza agents to minimize the mortality and morbidity associated with highly virulent strains in avian and human populations.

## Introduction

The avian influenza virus (AIV) H9N2 subtype was first characterized in 1966 as causing mild respiratory diseases in turkeys [1] and, for the first decade after its isolation, was found only in shorebirds and mallards [2]. However, after almost 50-years evolution and propagation, the H9N2 viruses have spread across most of the earth, circulating in wild bird and domestic poultry populations worldwide. Although AIV infections commonly result in low mortality in avian populations, in immunosuppressed chickens, recent isolates of H9N2 virus have been associated with high mortality in young chicks and a severe decline in egg production in laying chickens via secondary bacterial infections of the upper respiratory tract, resulted in significant economic losses of domestic poultry in many Asian countries [3-9]. In addition to the enormous financial losses, avian influenza virus (AIV) infections in bird populations with represent a repository of virulent genes or conservative gene fragments that can recombine to form new influenza A strains that can cross species boundaries, which increasingly are causing zoonotic infections in humans. One AIV subtype of increasing concern, H9N2, has spread across many countries with a broad range of host species, including humans, pigs and poultry [8-10]. For a longtime, H9N2 influenza virus was ignored because of its relatively low pathogenicity compared with H5N1. However, a recent zoonotic outbreak of H7N9 in China had resulted in many deaths, and the six internal genes (PA,NS,PB2,PB1,NP,M) of the new H7N9 strain come from H9N2 [11,12]. Similar involvement also announced before that some H9N2 and H1N1 hybrid can replicate in a high titer in vitro and exhibit higher pathogenicity than both parental viruses [12,13].

Sphingosine-1-phosphate receptor 1 (S1PR1) is one of the five sphingosine-1-phosphate (S1P) G protein-coupled receptors (GPCRs). The S1PR1 signaling system exerts its influence on a wide range of physiologic processes via activation of several signaling pathways: JAK/STAT, mTOR/PI3K/Akt, Ras/ERK/MAPK, Phospholipase C activation of PKC/Ca^2+^ and cyclic AMP [14,15]. Much research has been done on the role of on S1PR1 in cancer and neuroscience. S1PR1-STAT3 upregulation in tumor cells is critical for maintenance of persistent STAT3 signaling in cancer cells [16] and can result in premetastatic niche formation through activation of S1PR1-STAT3 and induced factors, enabling myeloid to invade and proliferate and apoptosis resistance at premetastic sites [17]. Protein S protects the blood–brain barrier integrity via Tyro3 and S1PR1, illuminating potentially novel treatments for neurovascular dysfunction resulting from blood–brain barrier damage [18]. However, until recently, little attention was given to the role of S1PR1 in immune response. In 2011, Teijaro, et al. used a S1P1 receptor subtype-selective agonist to suppress innate immune responses, resulting in suppression of cytokine and chemokine secretion and in significantly reduced mortality during infection of mice with a human pathogenic influenza H1N1 [19-21]. This indicated a crucial role of S1PR1 in influenza infection and suggested that S1PR1 signaling may be important in the immune response to other viral infections as well. The aim of the current study was to investigate the contribution of S1PR1 to infection with influenza virus by examining changes in the expression pattern of S1PR1, at the protein level and mRNA level, in normal (uninfected) mice compared to those infected with highly pathogenic and non-pathogenic H9N2 influenza. The results will contribute to get the function of S1PR1 in the infection of influenza virus and get the basic data for the strategies of anti-influenza.

## Results

### Amplification of S1PR1and sequence analysis

**Figure 1:**
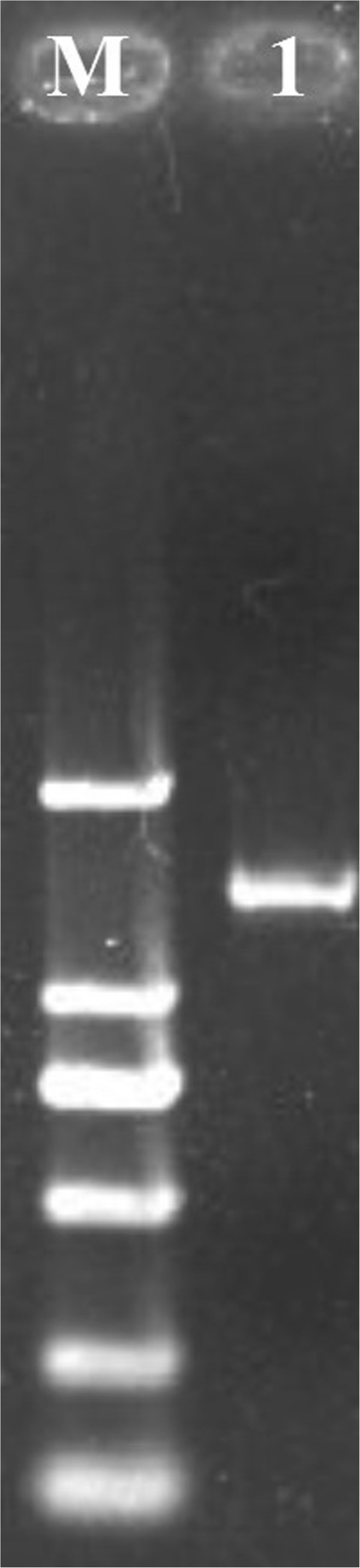
**Agarose gel electrophoresis of RT-PCR products of the S1PR1 of BALB/c mice.** M: Marker of DL2000; 1:S1PR1 gene (1483 bp).

The coding nucleotide sequence of S1PR1 gene of BALB/c was amplified by RT-PCR (Figure 1) and was analyzed by using the CLUSTAL W program (San Diego Supercomputer Center biology workbench: http://workbench.sdsc.edu/). It was revealed that the S1PR1 amplicon was approximately 1483 bp, which contained the complete coding sequence (1149 bp) of the S1PR1 gene, which was deposited in GenBank (accession no.: JX843822). The deduced protein sequence contains 382 amino acid residues, with a molecular mass of approximately 42 kDa. The nucleotide sequence of our cloned S1PR1 were aligned using the software MegAlign (DNAstar, Madison, WI) and were compared with similar sequences retrieved from the GenBank database (http://www.ncbi.nlm.nih.gov/, NCBI, US National Library of Medicine). Our cloned S1PR1 from BALB/c mice was 99%, 89%, 88%, 88% 87% and 86% identical with S1PR1 sequences previously cloned and submitted to GenBank for C57BL/6 J mice (RefSeq# NC_000069.6), horse (NC_009148.2), human (NM_001400.4), cattle (NM_001013585.4), sheep (NC_019458.1) and dog (NC_006588.3), respectively.

### mRNA expression of S1PR1 gene in BALB/c mice detected by Real-Time PCR

**Figure 2:**
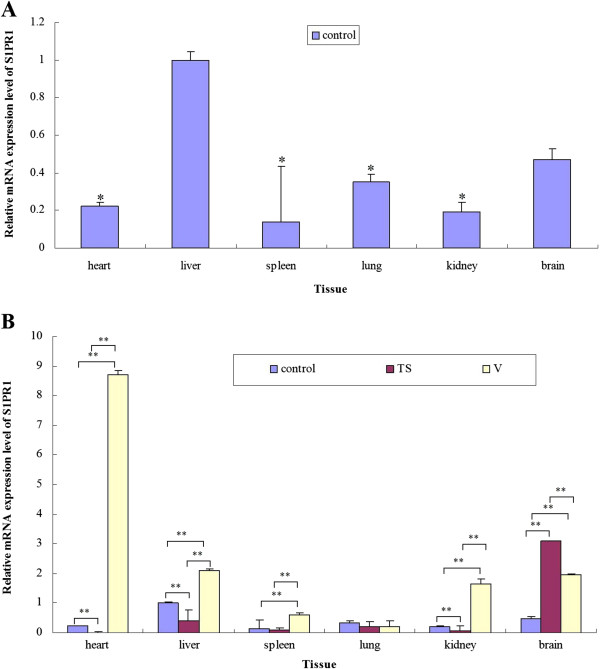
**Relative mRNA expression level of S1PR1 in the detected tissues of control, TS and V groups. A**, Relative expression levels of S1PR1 mRNA in control group; **B**, Differences in expression levels of S1PR1 mRNA between challenged group (TS- or V-) and the control group. ^*^, *P <* 0.05; ^**^, *P <* 0.01. Data are represented as mean ± standard deviation (SD). All samples were tested in triplicate.

The specificity of the S1PR1 gene and β-actin fragments obtained by PCR were confirmed by nucleotide sequences and by melting-curve profile analysis. In the normal BALB/c mice, S1PR1 mRNA was ubiquitously expressed in all the tissues examined, and the expression levels of S1PR1 was significantly lower in heart, spleen, lung and kidney tissues compared with liver (Figures 2A and B). There was no significant difference between S1PR1 expression levels in the liver and brain (Figure 2A). BALB/c mice challenged with the Ts strain demonstrated no observable symptoms of influenza infection in our previous reports [22,23]. However, when we examined the differences in mRNA expression levels between corresponding organs in negative control (unchallenged) BALB/c mice and BALB/c mice challenged with the TS strain, S1PR1 expression was significantly lower (*P* < 0.01) in heart, liver, kidney tissues (Figure 2B). However, levels of S1PR1 mRNA were significantly higher (*P* < 0.01) in the brain of TS-challenged mice compared to negative controls (3.10 ± 0.014 vs 0.47 ± 0.060) (Figure 2B). In comparison with BALB/c mice challenged with a highly virulent influenza strain (V), BALB/c mice challenged with the Ts showed significantly lower levels of S1PR1 mRNA in heart (*P* < 0.01), spleen (*P* < 0.01), kidney (*P* < 0.01), and liver (*P* < 0.05) (Figure 2B). However, mice in the TS-challenged group had significantly higher levels (*P* < 0.01) of S1PR1 mRNA in the brain than V-challenged mice (3.10 ± 0.014 vs 1.96 ± 0.049) (Figure 2B). The mRNA expression levels of S1PR1 in the heart, spleen, kidney and brain were significantly higher (*P* < 0.01) in V-challenged mice compared to negative controls (Figure 2B). No significant differences were seen in the S1PR1 mRNA levels of the lung among the three groups, and no significant differences were seen between mRNA levels in spleen of TS-challenged mice and negative controls (Figure 2B).

### S1PR1 expression detected by immunohistochemistry

**Figure 3:**
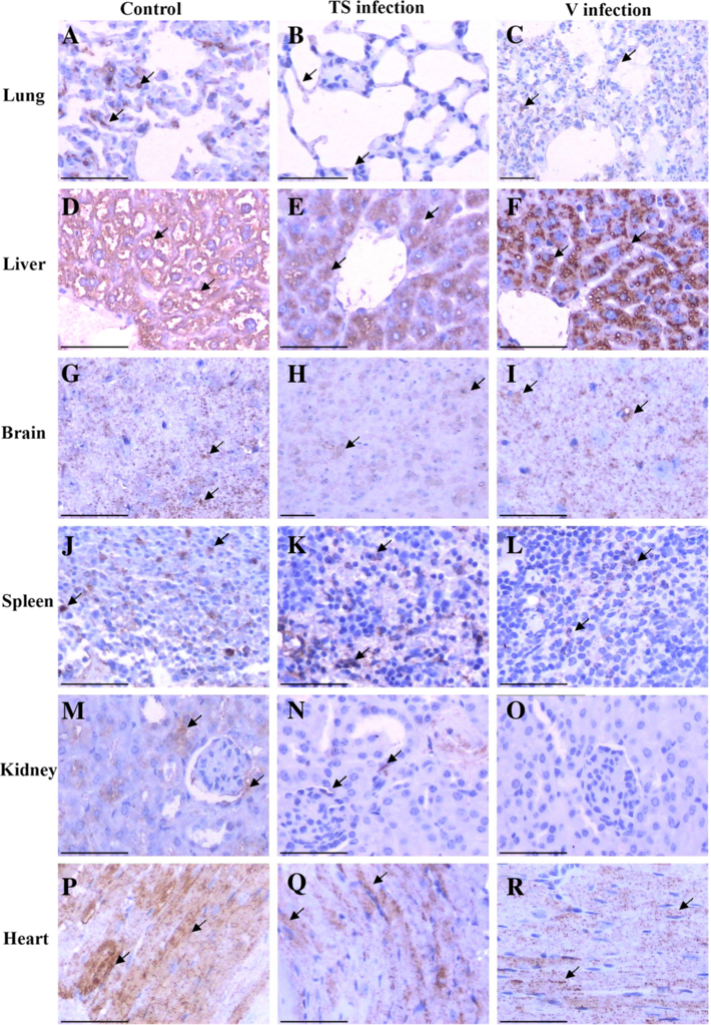
**Distribution of S1PR1 in the tissues of BALB/c mice.** Tissues were sectioned and immunohistochemical analysis was performed. Scale bar = 50 μm. **A**, **B** and **C**: lung of control, Ts and V group, respectively; **D**,**E** and **F**: Liver of control, Ts and V group, respectively; **G**,**H** and **I**: brain of control, Ts and V group, respectively; **J**,**K** and **L**: spleen of control, Ts and V group, respectively; **M**,**N** and **O**: kidney of control, Ts and V group, respectively; **P**, **Q** and **R**: heart of control, Ts and V group, respectively.

Immunohistochemical detection was performed to show the distribution of S1PR1 protein in the tissues of all three group mice (Figure 3). In the lung (Figures 3A, B and C), the epithelial cells the bronchi and bronchioles and the alveolar cells of the pulmonary alveoli were positive in the lung, and there is no significant difference between the three groups. In the liver (Figures 3D, E and F), hepatocytes were strongly positive in the cytoplasm but not in nucleus, and the endothelial cells of the hepatic blood vessels and sinus were negative. Overall staining of liver for S1PR1 protein in TS-challenged mice is lower than in V-challenged mice or the negative control group. In the brain (Figures. 3G, H and I), the S1PR1 showed positive staining in the cytoplasm of neurocytes and locally in the cells, and the amount of staining for the TS group is lower than for the control and V group. In the spleen (Figures 3J, K and L), only some splenocytes endothelial cells were positive, and this number is lower in the V group than in the control and TS group. In the kidney (Figures 3M, N and O), the endothelial cells of the some renal tubular and wall cell of glomeruli of kidney are stained diffusely for S1PR1 whereas the endothelial cells of the glomerulus were negative in normal control and TS group. There is no detection of S1PR1 expression in the kidney in V group (Figure 3O). Myocardial cells (Figures 3P, Q and R) were strongly positive in the normal control but it showed locally positive in the TS and V groups.

## Discussion

H9N2 subtype AIV has been circulating in domestic poultry worldwide after almost 50-years evolution and propagation. Though it is defined as a low pathogenic avian influenza itself, H9N2 has caused enormous losses of domestic poultry due to secondary infections, particularly in Asia and countries of the Middle East, H9N2 subtype AIV also represents a significant threat for novel zoonotic influenza viruses in humans via supplying virulent genes or conservative fragments to other influenza strains. Six of eight gene fragments of H7N9 influenza strains isolated in a recent outbreak of AIV in China were verified as being from H9N2 [12]. Moreover, recent reports have shown that the domestic cats and dogs were also susceptible to H9N2 AIV infection [24,25]; these species might pose an even bigger risk as a reservoir of zoonotic influenza virus due to their close proximity to humans in both developed and developing nations. Additionally, dogs may be able to transmit AIV strains between avian populations [25].

Inflammation is one of the common pathological features of viral infections, particularly in influenza infection, which is often characterized by excessive inflammation factors and cytokines, resulting in pathological damage of tissues and even fatalities in severe infections. Modulating inflammation during ongoing antiviral therapy to reduce resultant immunopathology is very likely to be beneficial and increase the chances to lower morbidity and mortality associated with some AIV strains [26]. Since the S1PR1 was first discovered on endothelial cells, it has been found engaging in a wide range of pathophysiological activities via modulating S1P signaling. S1PR1, by binding with S1P, could induce endothelial cell migration, proliferation, survival, and morphogenesis [27,28]. A precise S1P and S1PR1 signaling balance was found to be responsible for the cell growth and regulation of cell metabolism in mammals [29,30]. In addition, S1PR1 has been shown to control lymphocyte exit and to be required for the egress of both B and T cells [31,32]. However, an imbalance of S1P-S1PR1 system also participates in pathologic conditions such as cancer and inflammatory diseases [33,34].

It is because of S1PR1’s pivotal role in modulating the cellular inflammatory response that many researchers shifted their focuses to its role in inflammatory signaling in recent years. It has been demonstrated that over expression of S1PR1 along with other 4 S1P receptors will maintain a basal value even 7 days after injury, suggesting that S1PR1 activation may be a short-term injury response [35]. A recent investigation uncovered a novel function of S1PR1 as the critical regulator of efficient thrombopoiesis; mice lacking the S1P receptor S1PR1 developed severe thrombocytopenia, which suggests new therapeutic approaches for patients with this disease [36]. The role of S1PR1 in influenza infection has also been preliminarily studied by John R. Teijaro, et al. who suppressed excessive innate immune responses through S1P1 signaling, resulting in reduced mortality during infection of mice with a human pathogenic strain of influenza virus [20,21]. Hence, we hypothesized that S1PR1 might occupy a key position in influenza inflammation, protecting the host from excessive inflammatory symptoms caused by H9N2 infection, which we demonstrated in our previous reports [22,23]. Based upon our previous findings, we hypothesized that S1PR1 expression might be altered in mice challenged with two AIV strains associated with strong and weak inflammatory and related to differences in their respective mortality rates [21].

We examined the gene expression profile of S1PR1 to elucidate its function in influenza infection in mice challenged with H9N2 strains associated with high (V) and low (TS) pathogenicity. We found that S1PR1 mRNA was expressed in all tissues of BALB/c mouse examined; this may be the site-specific role of different tissues in infection. When using different influenza virus strains infected the BALB/c mice, the S1PR1 gene expression level showed different variation trends, we speculated this may be related to the strains of influenza virus and need to be further research. There were discrepancies, however, between the mRNA and protein expression levels in some tissues. Inconsistencies between mRNA and protein levels can be caused by many factors: post-transcriptional control, lags in protein translation, deactivation of post-translational protein processing, shorter half-lives of mRNA than proteins *in vivo*, and regulation of excessive mRNA by host molecules such as miRNA. We found significant differences in the expression pattern of S1PR1 mRNA or protein, both between uninfected and influenza-infected BALB/c mice and between those infected with H9N2 influenza strains that are non-pathogenic and highly pathogenic in mice (TS and V strains, respectively). These differences were most evident in tissues of heart, liver, kidney, brain. This difference correlates well with our previous reports, which identified differences in pathology in these organs between BALB/c mice infected with TS and V stains infection. Inflammation was more apparent in BALB/c mice infected with A/chicken/Guangdong/V/2008 (VK627), and this was confirmed by detection of increased expression of inflammatory cytokines [23]. Although our results confirm previous reports of the role of S1PR1 in pathology associated with infection with influenza, elucidation of the specific mechanism of S1PR1 in the inflammatory response to AIV infections requires further study.

## Conclusion

In conclusion, our data showing the distribution tissue expression pattern of S1PR1 in AIV-infected mice further supports a crucial role for S1PR1 in the physiological and pathological processes in influenza. The tissue-specific alteration in the expression pattern of S1PR1 in mice infected with highly pathogenic AIV compared with mice challenged with non-pathogenic AIV suggests differences in immune response are very likely to be associated with S1PR1 signaling pathways. Further research on S1PR1 signaling may enhance research and development of anti-influenza drugs and provide new opportunities for prevention and treatment of other human viral diseases.

## Materials and methods

### Viruses and BALB/c mice infection by H9N2 influenza virus

The viruses used in study were H9N2 AIV A/chicken/Guangdong/TS/2004 (TS_E627_), or TS, and A/chicken/Guangdong/V/2008 (V_K627_), or V. We previously determined that V virus replicates efficiently in mouse brains and is highly pathogenic in mice while TS-infected mice exhibit no clinical symptoms, and TS virus is not detectable in the brains of TS-infected mice [22,23]. Influenza viral titer was determined by plaque assay, as described in our previous study [22]. A total of 24 six-week old female mice (Guangdong Experimental Animal Center, Guangzhou, China) were randomly divided into 3 groups: TS group (infected by intranasal inoculation with 10^4^ PFU of TS influenza virus in 50 μl of sterile, endotoxin-free PBS); V group (infected intranasally with 10^4^ PFU of V influenza virus in 50 μl of sterile, endotoxin-free PBS); and Control group (treated intranasally with 50 μl of PBS alone). Mice were surveilled for daily weight loss, as a measure of morbidity, as our previous reports [22,23]. All animal experiments were conducted in a facility accredited by the Association for Assessment and Accreditation of Laboratory Animal Care International and in accordance with the guidance of CDC’s Institutional Animal Care and Use Committee. Additionally, all animal experiments had been authorized by Guangdong Province Animal Disease Control Center.

### Tissue preparation

All mice were anesthetized and euthanized at day 5 after inoculation. Tissues including heart, liver, spleen, lung, kidney, brain and stomach were harvested promptly. Each tissue was divided into two parts: one part was frozen in liquid nitrogen for 2 h then stored in -86°C until needed for RNA extraction; the other part was fixed in 10% neutralized buffered formalin for more than one week for immunohistochemistry.

### RNA extraction

Total RNA was extracted from 100 mg of frozen tissue from each organ using Trizol Reagent (Takara Biotech, Dalian, China), in accordance with the manufacturer’s instructions. After being mixed with trichloromethane and centrifuged, the supernatant was transferred and mixed with isopropanol and ethanol. Precipitated RNA was dissolved in RNAse-free H_2_O then treated with RNase-free DNase I to remove possible contaminating DNA before storage at -86°C.

### S1PR1 gene cloning

cDNA was generated from total RNA using reverse-transcriptase (Takara Biotech, Dalian, China), according to the manufacturer’s guidelines. Primers were synthesized as follows: 5’-TGCCTGGAGAAATACC-3’ (forward), 5’-TTCCCAGTGCATTGTT-3’ (reverse). The S1PR1 gene was amplified by polymerase chain reaction (PCR) in a 50 μl reaction volume with the following programmed conditions: Denaturation at 95°C for 5 min; 35 cycles (denaturation at 95°C for 30 s, annealing at 55°C for 40 s, and elongation at 72°C for 1 min); followed by a final elongation cycle at 72°C for 10 min. The cDNA produced by PCR were resolved by 1.5% agarose gel electrophoresis and visualized by ethidium bromide (EB) staining. Bands were purified by Gel Extraction Kit (Takara Biotech, Dalian, China). An aliquot of each purified PCR products were then cloned into pMD18-T vector (Takara Biotech, Dalian, China), and positive clones were sequenced by Takara Biotech company and were blast in pubmed.

### Real time quantitative PCR

The remaining purified cDNAs generated from selected tissues by PCR were normalized to the expression levels of the endogenous β-actin housekeeping gene Quantitative real-time PCR was performed in a total 25 μl reaction volume containing 2 μl of total cDNA, 0.5 μl of forward and reverse primers (20 μM each), 22 μl SYBR^®^ Premix Ex Taq™ reaction mixture (Takara Biotech, Dalian, China) using Bio-Rad iQ™5 Real-Time PCR Detection System (Applied Biosystems, Carlsbad, CA). The primers for S1PR1 gene fragment and β-actin are as follows: S1PR1: 5′-TGCCTGGAGAAATACC-3′ (forward), 5′-TTCCC AGTGCATTGTT-3′ (reverse), β-actin: 5′-CATCCGTAAAGACCTCTATGCCAAC-3′ (forward), 5′-ATGGAGCCACCGATCCACA-3′ (reverse). The reaction was carried out with the following programmed parameters: 35 cycles (95°C for 30 s, 55°C for 40 s, and 72°C for 30 s); followed by a final elongation cycle at 72°C for 10 min. All reactions were performed in triplicates. The relative expression level of S1PR1 gene was calculated by normalizing the levels of S1PR1 transcripts to that of β-actin transcripts using a relative standard curve method. The expression diversity of the S1PR1 gene was transformed using the 2^-ΔΔCt^ formula and normalized with β-actin expression. The melting curves for each PCR were carefully analyzed to avoid nonspecific amplifications in PCR products. Data was analyzed using GraphPad Prism 5 (GraphPad Software, La Jolla, CA). Independent sample (two-tailed) *t*-test and one way anove was used to determine statistical significance between samples. A p-value less than 0.05 (*P* < 0.05) was considered to be statistically significant, while a p-value less than 0.01 (*P* < 0.01) was considered to be highly significant.

### Immunohistochemical detection

Samples of formalin fixed tissues were trimmed, dehydrated, embedded in melted paraffin, sliced into 4 μm thick sections and finally mounted on silanized slides pre-treated with silicon. Endogenous peroxidase activities in tissue sections were inactivated in 3% H_2_O_2_ for 10 min at room temperature and washed 3 times by 0.02 M PBS (pH = 7.2) at 5 min intervals. Heat-induced epitope retrieval was performed by immersing tissue sections in 10 mM sodium citrate pH 6.0 at high pressure, 100°C for 10 min and then cooled naturally. After being washed 3 times in PBS for 5 min, sections were incubated with rabbit anti-S1PR1 polyclonal antibody (PROTEINTECH, USA), diluted 1:100, for 1.5 h at 37°C. Sections were again washed 3 times in PBS then incubated with HRP-conjugated goat anti-rabbit IgG antibody for 1 h at 37°C. S1PR1 was then visualized by DAB (3, 3’-diaminobenzidine-tetrahydrochloride) for 4 min according to the manufacturer’s guidelines (ZSGQ-BIO, China). Tissues were dyed using hematoxylin for 1 min, washed under running water for 10 min, dehydrated with histologic grade ethanol, vitrified by dimethylbenzene, and, finally, embedded in neutral balsam.

## Competing interests

The authors declare no conflict of interest.

## Authors’ contributions

Conceived and designed the experiments: ML and ZN. Performed the experiments: ST, TJ, HW, ZH and MY. Analyzed the data: HW, LS and RL. Wrote the paper: ST, TJ and ZN. All authors read and approved the final manuscript.
